# The anticarcinogenic effect of eugenol on lung cancer induced by diethylnitrosamine/2-acetylaminofluorene in Wistar rats: insight on the mechanisms of action

**DOI:** 10.1007/s10495-023-01852-2

**Published:** 2023-05-13

**Authors:** Hadeer M. Morsy, Osama M. Ahmed, Khairy M. A. Zoheir, Adel Abdel-Moneim

**Affiliations:** 1 Physiology Division, Zoology Department, Faculty of Science, Beni-Suef University, P.O. Box 62521, Beni-Suef, Egypt; 2 Cell Biology Department, Biotechnology Research Institute, National Research Centre, Cairo, 12622 Egypt

**Keywords:** Lung cancer, Diethylnitrosamine, Acetylaminofluorene, Eugenol, Inflammation, Apoptosis

## Abstract

This study was designed to assess the ameliorative effects of eugenol and to propose the possible mechanisms of action of eugenol in diethylnitrosamine (DENA)/acetylaminofluorene (AAF)-caused lung cancer in Wistar rats. To induce lung cancer, DENA at a dose of 150 mg/kg body weight (b.wt) for 2 weeks were intraperitoneally injected once each week and AAF was administered orally at a dose of 20 mg/kg b.wt. four times each week for the next 3 weeks. DENA/AAF-administered rats were orally supplemented with eugenol at a dose of 20 mg/kg b.wt administered once a day until 17 weeks starting from the 1st week of DENA administration. Lung histological lesions, including sheets of tumor cells, micropapillary adenocarcinoma, and apoptotic cells, resulting from the DENA/AAF dosage, were ameliorated by eugenol treatment. However, a significant drop in the levels of LPO in the lungs and a remarkable rise in GSH content and GPx and SOD activities were observed in DENA/AAF-administered rats treated with eugenol compared with those in DENA/AAF-administered controls. Moreover, in DENA/AAF-administered rats, eugenol supplementation significantly reduced TNF-α and IL-1β levels and mRNA expression levels of NF-κB, NF-κB p65, and MCP-1 but significantly elevated the level of Nrf2. Furthermore, the DENA/AAF-administered rats treated with eugenol exhibited a significant downregulation of Bcl-2 expression levels in addition to a significant upregulation in P53 and Bax expression levels. Otherwise, the administration of DENA/AAF elevated the protein expression level of Ki-67, and this elevation was reversed by eugenol treatment. In conclusion, eugenol has effective antioxidant, anti-inflammatory, proapoptotic, and antiproliferative properties against lung cancer.

**Graphical abstract:**

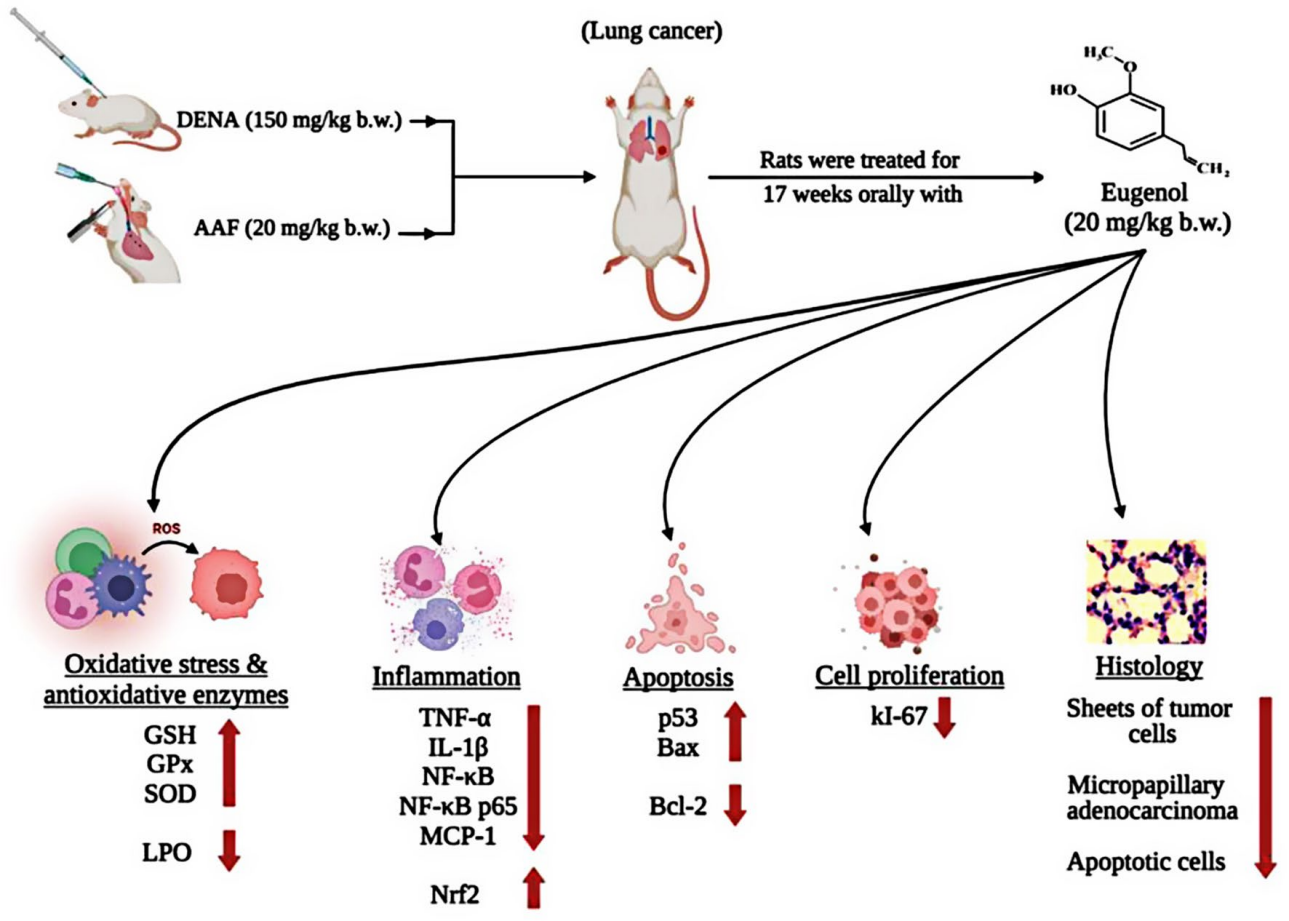

## Introduction

Lung cancer is one of the leading causes of cancer-related death worldwide [1]. This disease is distinguished by unregulated cell proliferation in lung tissues [2]. Worldwide, 2,094,000 new patients with lung cancer were detected in 2018, according to the most recent GLOBOCAN estimation, keeping lung cancer the most common cancer downfall globally [3, 4]. Environmental and genetic danger indicators are the most important inducers of lung cancer. Tobacco smoking represents the greatest risk factor for the advancement of lung cancer, accounting for up to 90% of all lung cancer cases [5]. Hence, innovative treatments should be intended and severely estimated in the battle against this fatal malignancy. Diethylnitrosamine (DENA) is a chemical carcinogen that acts as a cancer initiator in various organs [6]. In Wistar rats, it triggered lung cancer [7]. It can be used alone or along with a cancer promoter, such as acetylaminofluorene (AAF) to stimulate cancer cell formation, to produce cancer cells [8].

The progression of carcinogenesis is affected by oxidative stress, inflammation, and apoptosis [9, 10]. Oxidative stress is important in the initiation, progression, and invasiveness of lung cancer [11]. Reactive oxygen species (ROS) are reported to cause DNA damage and mutations in purines, pyrimidines, and oxidate chromatin, causing genomic instability, influencing gene expression, and promoting carcinogenesis and cancer progression [12]. Otherwise, the stimulation of many transcription factors by oxidative stress, resulting in the differential expression of many genes associated with inflammatory pathways, causes several chronic diseases [13]. Inflammation has both pro- and antitumor properties. Inflammation can activate immune cells and cause the release of cytokines, which can prevent tumor growth. However, inflammation may influence cancer initiation, progression, and metastasis [14]. Apoptosis is most commonly activated during cancer cell death due to multiple drug treatments [15]. The apoptotic pathways in cancer, including intrinsic and extrinsic pathways, are often suppressed through numerous mechanisms, including antiapoptotic protein overexpression and proapoptotic protein under-expression [10]. The interaction of these apoptotic pathways with other signaling mechanisms can also affect cell death [16].

Many medicinal plants used in traditional medicine are known as significant sources of natural antioxidants [17–19]. These plants attract more attention for their efficiency against several diseases such as cancer, atherosclerosis and diabetes [20–23]. Natural antioxidants are very efficient in blocking the process of oxidation by neutralizing free radicals and activated oxygen species [17, 24]. The antioxidative properties of medicinal plants are related to the presence of natural antioxidants such as phenolics which are found in clove [25]. Phenolic antioxidants have pharmacological actions which stem primarily from their metal chelating and free radical scavenging properties in conjunction with their effects on gene expression and cell signaling pathways [26]. Eugenol, a natural phenolic compound derived from clove oil and lignin depolymerization, has a chemical composition that enables facile modulation to create a varied and multilateral bio-based monomer platform [27]. This compound has been investigated extensively and has been demonstrated to have various biological processes, such as antimicrobial, antifungal, antioxidant, anti-inflammatory, anticancer, analgesic, antiparasitic [28], antitumor [29], and antibacterial influences [30].

Remarkably, no research has reported the effects of eugenol on DENA/AAF-enhanced rats with lung cancer. Consequently, this investigation was designed to evaluate the putative chemopreventive effect of eugenol on DENA/AAF-induced lung cancer and to postulate its possible action mechanisms.

## Materials and methods

### Experimental animals

In this investigation, male Wistar rats weighing 100–120 g were included. The Egyptian Organization for Biological Products and Vaccines, Helwan Station, Cairo, Egypt, delivered the animals. To eliminate the risk of chronic infections, the animals were kept under observation for 14 days prior to the outset of the experiment. The rats were kept in well-ventilated polypropylene cages at the Zoology Department, Faculty of Science, Beni-Suef University, Egypt, at a regular daily lighting cycle (10**–**12 h/day), normal temperature (20**–**25 °C), and adequate food and drink have been provided. The Experimental Animal Ethics Committee of the Faculty of Science for the Care and Use of Animals, Beni-Suef University, Egypt (ethical approval number: BSU/FS/2019/3) has confirmed and approved all experimental steps and the research strategy. All measures were performed to diminish the number of animals and to reduce animal anguish, pain, and discomfort.

### Chemicals

DENA (#049K1613V), AAF (WZDQE-ED), eugenol (#STBH9243), and carboxymethyl cellulose (CMC) (I024086) were purchased from Sigma-Aldrich Chemicals Co. (St. Louis, MO, USA) and were preserved at 2**–**4 °C, except for eugenol and CMC, which were stored at room temperature.

### Experimental protocol

**Fig. 1:**
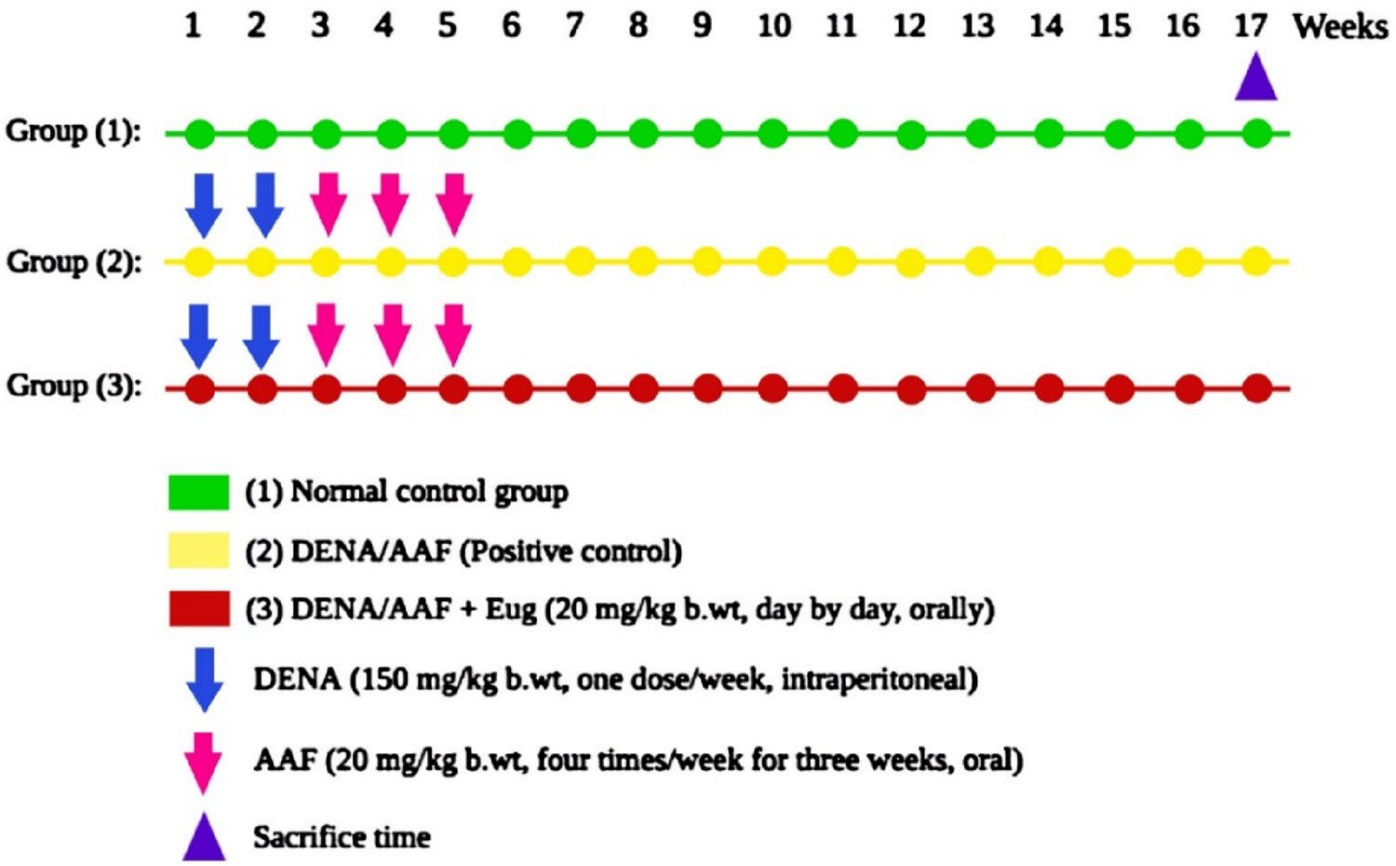
Experimental design for the administration of diethylnitrosamine/acetylaminofluorene, and eugenol

Thirty male mature Wistar rats were subdivided into three groups, with 10 animals in each group. Group I was used as the standard control (normal control group). However, the two other groups received intraperitoneal injections of DENA at a dose of 150 mg/kg body weight (b.wt) for 2 weeks, once each week, followed by oral AAF at a dose of 20 mg/kg b.wt for 3 weeks, four times each week [31]. Group II was given DENA/AAF and was used as the positive control group, and Group III comprised DENA/AAF-administered rats treated with oral eugenol at a dose of 20 mg/kg b.wt [32] dissolved in 1% CMC, administered every other day for 17 weeks starting from the 1st week of DENA administration (Fig. 1).

### Blood and lung sampling

After 119 days, after drugging the animals, blood samples were taken from the jugular vein under mild anesthesia after diethyl ether inhalation, and following decollation, lung tissue samples were collected for molecular, biochemical, and histopathological studies. After allowing the blood samples to clot at room temperature, they were centrifuged at 3000 rpm for 15 min. The supernatant sera were collected in sterile containers and stored at − 20 °C using a Pasteur pipette. Then, 1 g of lung tissue was frozen and thawed and washed with ice-cold water. By homogenizing NaCl solution (0.9%) in 10-mL 0.9% NaCl, 1% of homogenate (w/v) was obtained. For 15 min, lung homogenates were centrifuged at 3000 rpm. Then, 3 mm^3^ lung pieces were maintained in sterilized Eppendorf tubes at − 70 °C until they were used for real-time polymerase chain reaction (RT-PCR) analysis and RNA isolation.

### Detection of oxidative stress and antioxidant defense system

The spectrophotometric kits of lipid peroxidation (LPO) (MD 25 28), glutathione (GSH) (GR 25 10) content, and antioxidant enzyme activities, such as superoxide dismutase (SOD) (SD 25 20) and glutathione peroxidase (GPx) (GP 2524), were obtained from Biodiagnostic co. (Giza, Egypt). The detections were carried out in lung homogenate supernatants according to the manufacturer’s instructions.

### Detection of lung inflammatory biomarkers

Tumor necrosis factor-α (TNF-α) was assayed using the quantitative enzyme-linked immunosorbent assay (ELISA) technique using kit (CSB-E11987r) delivered from R&D Systems (USA); interleukin-1β (IL-1β) level was estimated using the ELISA kit (# MBS825017) obtained from Ray Biotech (USA). Moreover, the transcriptional activity of nuclear factor-erythroid factor 2-related factor 2 (Nrf2) was measured using the ELISA kit (MBS752046) obtained from Abcam, Cambridge, UK. All determinations of inflammatory biomarkers were according to the manufacturer’s instructions.

### Western blotting of Ki-67

Western blotting analysis was performed for lung Ki-67, as previously claimed by Tawfik et al. [33]. Briefly, lung samples were homogenized in radioimmunoprecipitation assay buffer, and supernatants were acquired by centrifugation. The total protein concentration was measured by using the Bradford reagent. Sodium dodecyl-sulfate polyacrylamide gel electrophoresis was used to isolate 30-µg protein per gel lane, which was then transferred to nitrocellulose membranes. After blocking membranes in Tris-buffered saline with Tween 20 (TBST) containing 5% non-fat milk powder, they were incubated with primary antibodies against Ki-67 (EMD Millipore, Burlington, MA, USA) and β-actin (Santa Cruz Biotechnology, Inc., Dallas, TX, USA). The housekeeping protein β-actin was employed as a loading control to standardize the protein levels detected by demonstrating that protein loading is consistent across the gel. After washing with TBST, the membranes were incubated with horseradish peroxidase-conjugated secondary antibodies (Novus Biologicals, Littleton, CO, USA) for 1 h. An enhanced chemiluminescence kit (BioRad, Hercules, CA, USA) was used to determine immunolabeling. Lastly, the blots were scanned and band intensities were quantified using ImageJ (NIH, Bethesda, MD, USA).

### RNA isolation and quantitative qRT-PCR analysis

Total RNA was isolated from lung tissues using the TRIzol reagent (Invitrogen), following the manufacturer’s instructions. Isolation was performed by calculating the 260-nm absorbance; the 260:280 ratio was used to evaluate the RNA quality. The High-Capacity cDNA Reverse Transcription kit (A32702) (Applied Biosystems) was used to synthesize complementary DNA (cDNA). Glyceraldehyde 3-phosphate dehydrogenase (GAPDH) was used as the internal control. The following quantitative studies of the mRNA expression of target genes were conducted using RT-PCR in the ABI Prism 7500 System (Applied Biosystems): A cDNA synthesis kit (Thermo-Fisher Scientific) was used to transform 1-g RNA into cDNA. The primer sequences for the genes screened in this investigation are shown in Table 1. The QuantStudio™ 7 Flex Real-Time PCR System (Thermo Scientific) was used to run RT-PCR with PowerUp™ SYBR^®^ Green Master Mix (Thermo-Fisher Scientific). GAPDH was used as the internal control. The gene relative expression was estimated using the 2^−∆∆CT^ technique. No. 2 User Bulletin. In brief, the findings were shown as the gene expression fold change compared with a calibrator and adjusted to the endogenous reference gene (GAPDH). The primers’ pair sequences (forward and reverse) were used in this investigation (Table 1).

**Table 1:** Sequence of the primers used in the experiment

mRNA species	GenBank accession number	Primer sequence 5′–3′
NF-κB	NM_001276711.1	F: 5′ TTCAACATGGCAGACGACGA3′ R: 5′ TGCTCTAGTATTTGAAGGTATGGG3′
NF-κB p65	NM_001145138.2	F: 5′ TCAAGACAGATCAGAAGCGA3′ R: 5′ TACCTGAGTGTCTGGGATGG3′
MCP-1	NM_002982	F: 5′ ATGCAGTTAATGCCCCACTC3′ R: 5′ TTCCTTATTGGGGTCAGCAC3′
P53	AH010014.2	F: 5′ GTTTTTGTTCCTGAGCCCCG3′ R: 5′ GAGCAAGGGGTGACTTTGGG3′
Bcl-2	NM_016993.2	F: 5′ GGGGCTACGAGTGGGATACT3′ R: 5′ GACGGTAGCGACGAGAGAAG3′
BAX	NM_017059.2	F: 5′ AGACACCTGAGCTGACCTTG3′ R: 5′ GTTGTTGTCCAGTTCATCGCC3′
GAPDH	NM_017008.4	F: 5′ GCGAGATCCCGCTAACATCA3′ R: 5′ ATTCGAGAGAAGGGAGGGCT3′

NF-κB; Nuclear factor kappa-light-chain-enhancer of activated B cells, NF-κB p65; NF-kappa B p65, MCP-1; monocyte chemoattractant protein-1, P53; tumor suppressor p53, Bcl-2; B-cell lymphoma 2, BAX; Bcl-2-associated X protein, GAPDH; Glyceraldehyde-3-Phosphate Dehydrogenase

### Histopathological investigations

After 119 days, after dissecting, each rat’s lung was removed. Small lung sections from all groups of rats were fixed in 10% neutral buffered formalin for 24 h. Then, all sections were cleaned and embedded in paraffin at 56 °C in a hot air oven for 24 h before being added to 70% alcohol for histological examination. At 5 microns, paraffin wax tissue blocks were ready for cutting. Hematoxylin and eosin were used to stain the tissue sections [34]. A light microscope with a camera was used for the examination.

### Statistical analysis

The obtained data were analyzed with the Statistical Package for the Social Sciences (SPSS), version 20 (IBM Corp., Armonk, NY, USA). The results were presented as means ± standard errors. Duncan's post-hoc analysis technique was used for all statistical comparisons. Statistical significance was denoted by *p* values less than 0.05.

## Results

### Effects on lung oxidative stress and the antioxidant defense system

The LPO levels were significantly increased (*p* < 0.05) and the GSH content and GPx and SOD activities were decreased significantly (*p* < 0.05) in DENA/AAF-treated rats compared with those in the normal control rats. DENA/AAF-administered rats treated with eugenol significantly prevented the increase in the LPO level and the decrease in the GSH content and GPx and SOD activities (*p* < 0.05) compared with DENA/AAF-treated controls (Table 2).

**Table 2:** Effect of eugenol on lung LPO level, GSH content and SOD, and GPx activities in DENA/AAF-administered rats

Groups	LPO (ng/mg protein)	GSH (mmol/mg protein)	GPx (mmol/mg protein)	SOD (U/mg protein)
Normal	72.26 ± 2.38^a^	95.36 ± 0.60^c^	204.56 ± 3.85^b^	16.63 ± 1.18^c^
DENA/AAF	101.70 ± 1.71^b^	47.23 ± 1.11^a^	87.63 ± 1.98^a^	3.33 ± 0.18^a^
DENA/AAF + Eug	70.96 ± 7.40^a^	64.26 ± 6.17^b^	200.66 ± 3.14^b^	9.10 ± 0.42^b^

The results are presented as means ± standard errors. Each group has six samples that have been detected. Values with various superscript symbols (a, b, and c) for each parameter differ significantly at < 0.05

*LPO* lipid peroxidation, *GSH* glutathione, *GPx* glutathione peroxidase, *SOD* superoxide dismutase, *DENA*/*AAF* diethylnitrosamine/acetylaminofluorene, *Eug* eugenol

### Effects on lung inflammation

**Fig. 2:**
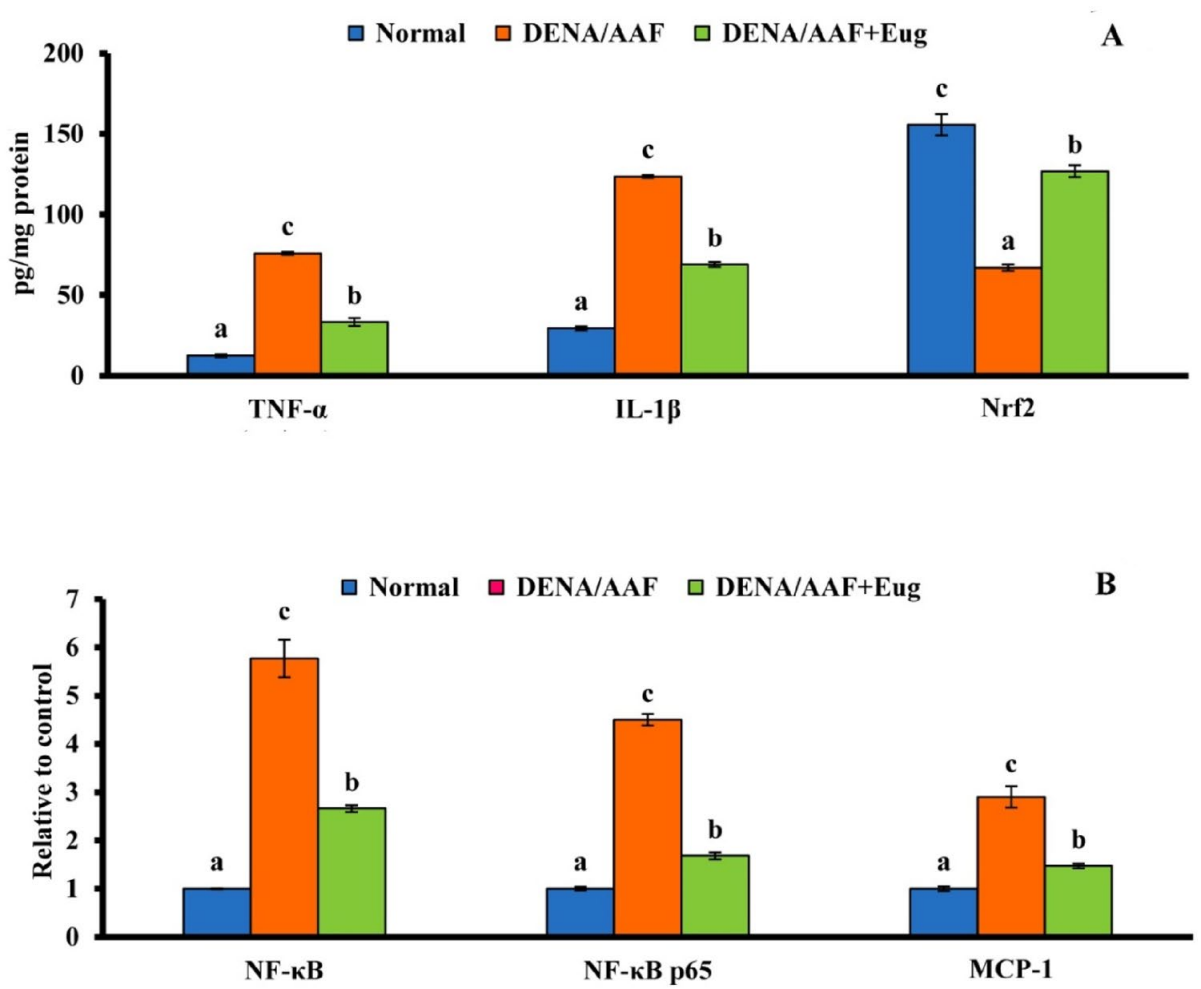
Effect of eugenol on lung TNF-α; IL-1β, and Nrf2 levels (**A**) and NF-κB, NF-κB P65, and MCP-1 mRNA expression levels (**B**) in DENA/AAF-administered rats. The results are presented as means ± standard errors. Each group has six samples that have been detected. Values with various superscript symbols (a, b, and c) for each parameter differ significantly at < 0.05. TNF-α, tumor necrosis factor alpha; IL-1β, interleukin-1beta; Nrf2, nuclear factor-erythroid factor 2-related factor 2; NF-κB, nuclear factor kappa-light-chain-enhancer of activated B cells; NF-κB p65, nuclear factor kappa-light-chain-enhancer of activated B cells p65; MCP-1, monocyte chemoattractant protein-1; DENA/AAF, diethylnitrosamine/acetylaminofluorene

The administration of DENA/AAF significantly increased the lung TNF-α and IL-1β levels and mRNA expression levels of NF-κB, NF-κB p65, and MCP-1 (*p* < 0.05) and significantly reduced Nrf2 levels (*p* < 0.05) compared with the normal control group. Moreover, eugenol supplementation to DENA/AAF-administered rats significantly reduced TNF-α and IL-1β levels and NF-κB, MCP-1, and NF-κB P65 mRNA expression levels (*p* < 0.05) and significantly increased Nrf2 levels (*p* < 0.05) (Fig. 2).

### Effects on lung apoptosis

**Fig. 3:**
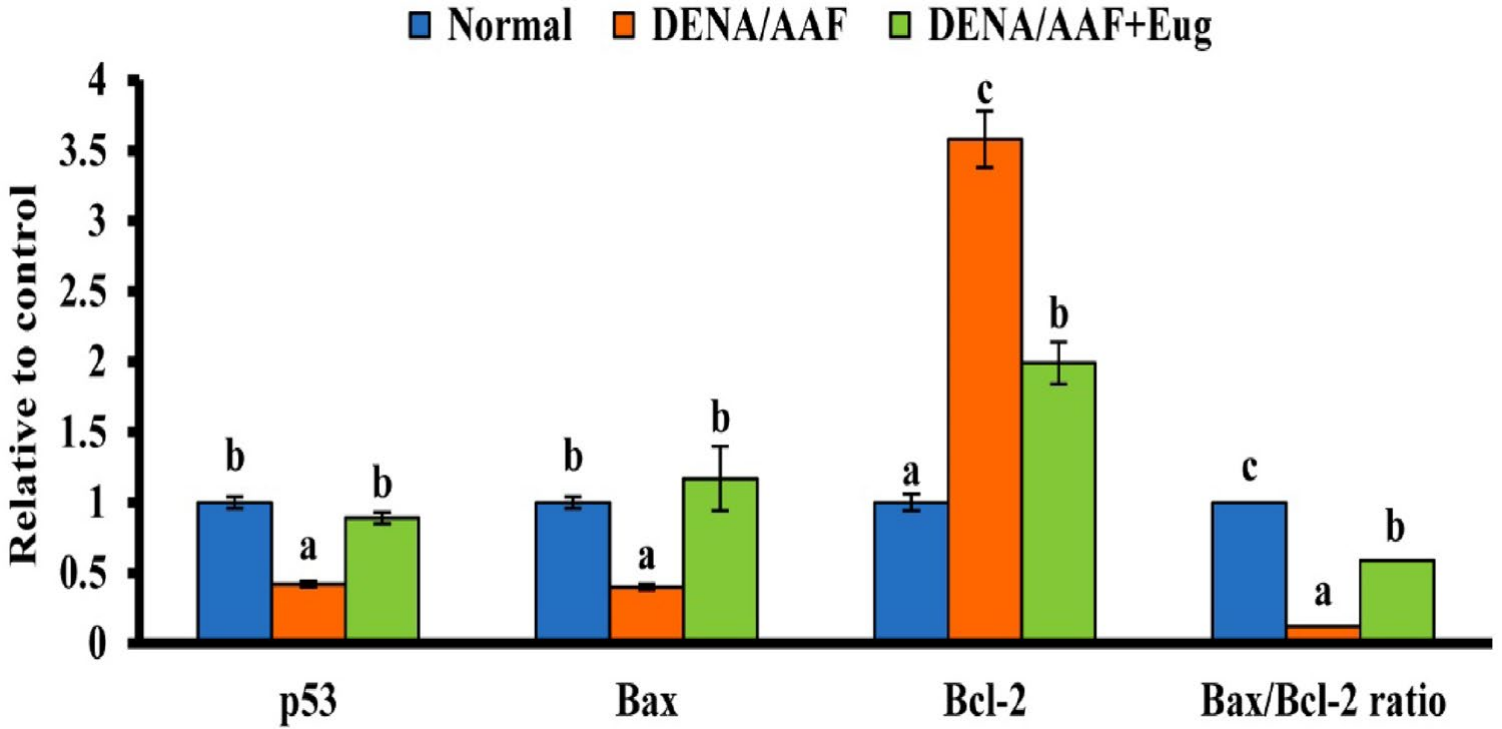
Effect of eugenol on lung p53, Bax, and Bcl-2 expression levels and the Bax/Bcl-2 ratio in DENA/AAF-administered rats. The results are presented as mean ± standard errors. Each group has six samples that have been detected. Values with various superscript symbols (a, b, and c) for each parameter differ significantly at < 0.05. Bcl-2, B-cell lymphoma 2; p53, tumor suppressor gene 53; Bax, Bcl-2-associated X protein

The data revealed that the administration of DENA/AAF significantly decreased mRNA expression of p53 and Bax in the lungs and Bax/Bcl-2 ratio (*p* < 0.05) and increased significantly mRNA expression level of Bcl-2 (*p* < 0.05) relative to the normal control group. In contrast, eugenol supplementation to DENA/AAF-administered rats significantly (*p* < 0.05) increased mRNA expression of p53 and Bax and Bax/Bcl-2 ratio and reduced the expression level of Bcl-2 relative to DENA/AAF-administered control rats (Fig. 3).

### Effect on lung cell proliferation and division

**Fig. 4:**
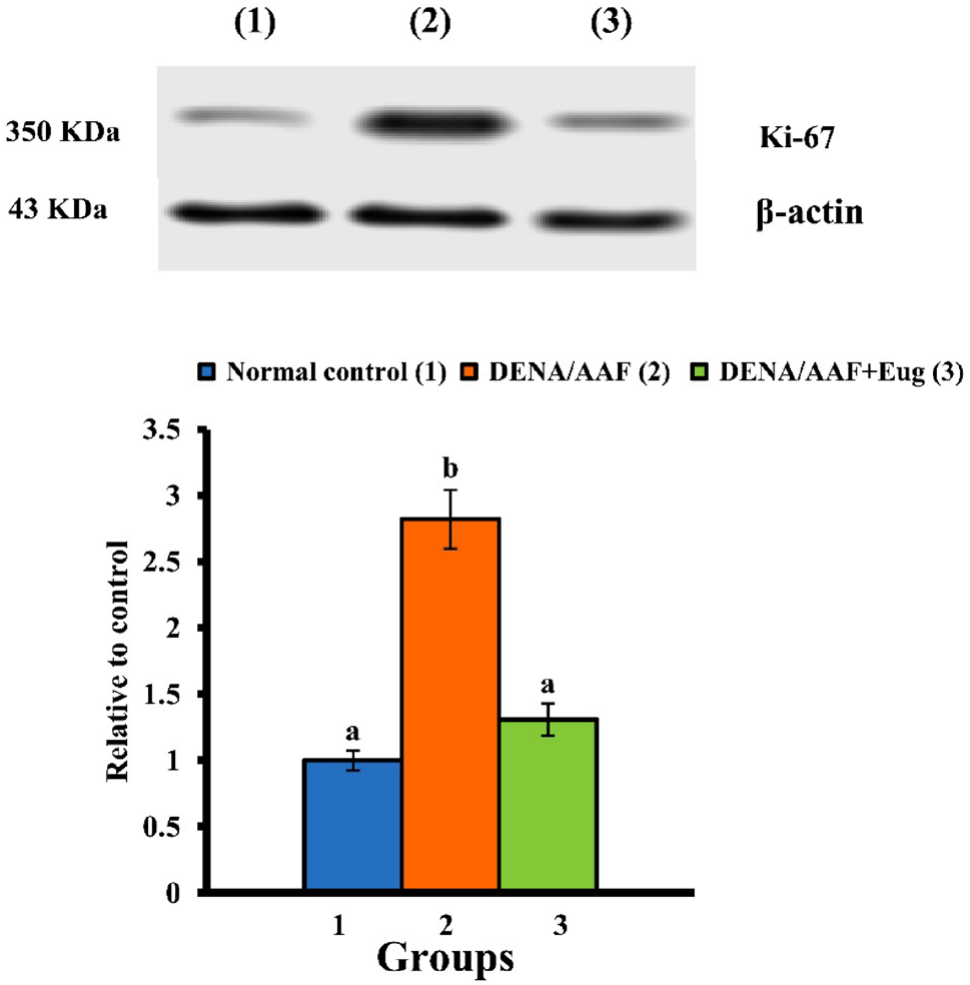
Effect of eugenol on lung Ki-67 level in DENA/AAF-administered rats. The results are presented as means ± standard errors. Each group has six samples that have been detected. Values with various superscript symbols (a, b, and c) for each parameter differ significantly at < 0.05

The administration of DENA/AAF significantly upregulated Ki-67 expression levels (*p* < 0.05) relative to the normal control group. Moreover, eugenol treatment significantly downregulated Ki-67 expression levels (*p* < 0.05) (Fig. 4).

### Histopathological effects

**Fig. 5:**
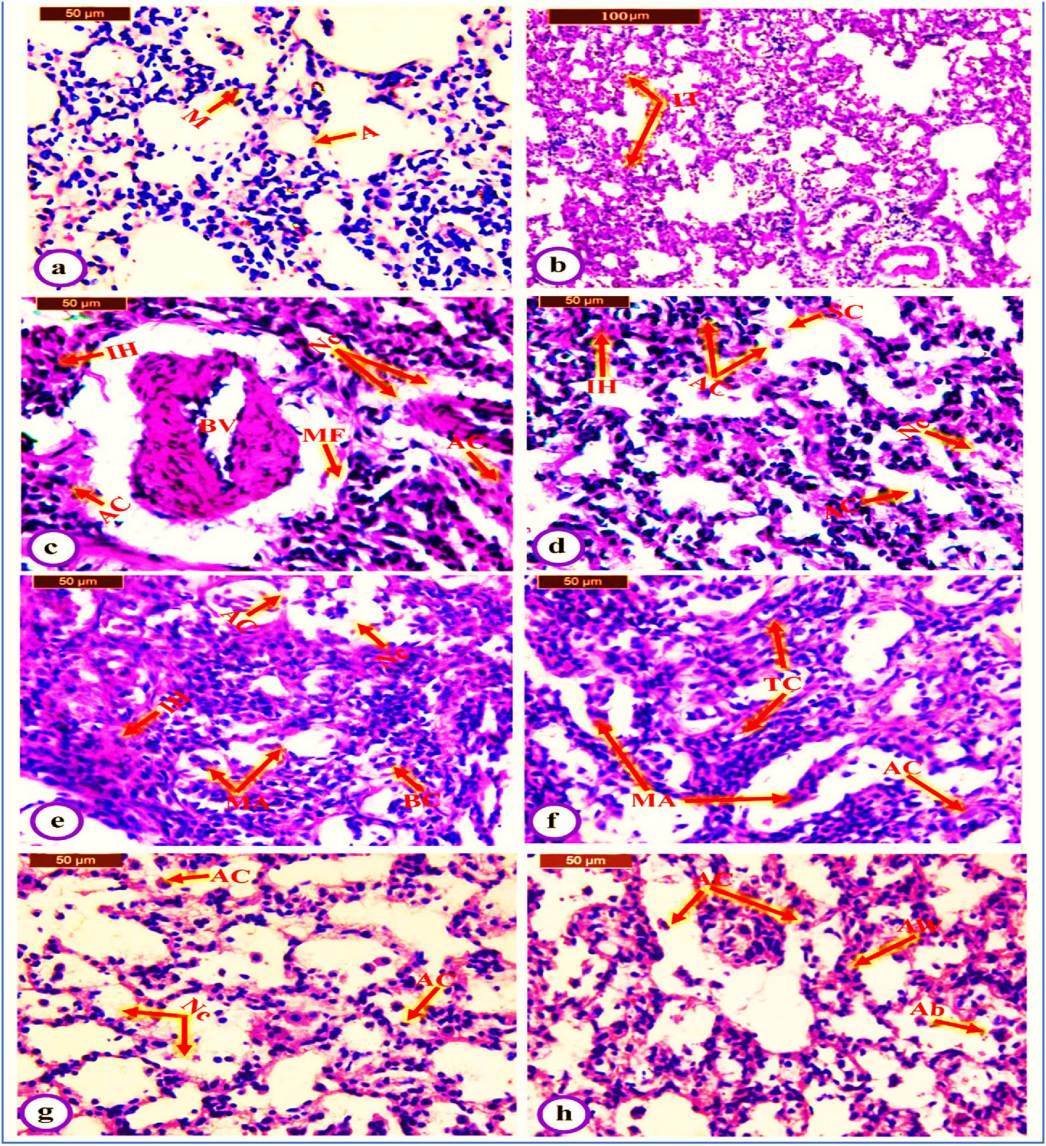
Photomicrographs (a × 400) of the normal lung structure showing normal histology in the alveolar area (A) and occasional pulmonary macrophages (M), DENA/AAF-administered rats (b, × 100 and c–f, × 400) demonstrated several lung cancerous changes, such as diffuse thickening in interstitial tissue (IT), micropapillary adenocarcinoma (MA), sheets of tumor cells (TC), severely degenerated blood vessels (BV), mononuclear leukocytic infiltration (MF), necrosis (Nc), interstitial hemorrhage (IH), binucleated cells (BC), and the sloughing of cells into the alveoli (SC) and apoptotic cells with eosinophilic cytoplasm and apoptotic bodies (AC). DENA/AAF-administered rats treated with eugenol (g and h, × 400) showing normal lung architecture with alveolar walls with fibrous thickening (AW); most appeared to be normal, and some showed necrosis (Nc), apoptotic cells (AC), and apoptotic blebs (Ab)

The histopathological examination was designed to investigate the influence of eugenol on the integrity and architecture of lung tissues in DENA/AAF-administered rats (Fig. 5). Histologically, the lung tissues of normal control rats appeared normal in the alveolar area with thin and delicate walls; the alveoli were well-aerated and only contained occasional pulmonary macrophages (Fig. 5a). Moreover, the administration of DENA/AAF causes lung histological changes, including diffuse thickening in the interstitial tissue (Fig. 5b), severely degenerated blood vessels, collapsed alveoli with other compensatory alveoli separated by a thickened inter-alveolar septum, infiltration of mononuclear leukocytes into the parenchyma (Fig. 5c), diffuse thickening of interstitial tissue, interstitial hemorrhage, sloughing of cells into alveoli, apoptotic cells with apoptotic bodies (Fig. 5d), micropapillary adenocarcinoma consisting of little papillary clusters of glandular cells that form in the airspace (Fig. 5e), acinar glands that invade the fibrous stroma, tumor cell sheets with plentiful cytoplasm and predominantly vesicular nuclei with many obvious nucleoli, apoptotic cells with eosinophilic cytoplasm, and condensed chromatin in the nucleus (Fig. 5f). In contrast, DENA/AAF-administered rats treated with eugenol exhibited significantly improved lung histological architecture and integrity. However, they showed some alveolar walls with fibrous thickening, and most rats displayed normal and apoptotic cells with eosinophilic cytoplasm and condensed chromatin in the nucleus; some apoptotic blebs were also observed (Fig. 5g, h). The histological results of this investigation, determined by histopathological evaluations of lung lesions obtained from the three groups, are shown in Table 3.

**Table 3:** Histopathological evaluations of lung lesions in normal control, DENA/AAF-administered group and DENA/AAF-administered group treated with eugenol

Histopathological changes	Score	Normal	DENA/AAF	DENA/AAF + Eug
Inflammation	0	6 (100%)	**–**	4 (66.6%)
	I	**–**	3 (50%)	2 (33.3%)
	II	**–**	2 (33.3%)	**–**
	III	**–**	1 (16.7%)	**–**
Necrosis	0	6 (100%)	**–**	4 (66.6%)
	I	**–**	2 (33.3%)	1 (16.7%)
	II	**–**	2 (33.3%)	1 (16.7%)
	III	**–**	2 (33.3%)	**–**
Vascular congestion	0	6 (100%)	4 (66.6%)	5 (83.3%)
	I	**–**	1 (16.7%)	1 (16.7%)
	II	**–**	1 (16.7%)	**–**
	III	**–**	**–**	**–**
Tumor cells	0	6 (100%)	**–**	3 (50%)
	I	**–**	1 (16.7%)	2 (33.3%)
	II	**–**	3 (50%)	1 (16.7%)
	III	**–**	2 (33.3%)	**–**
Cytoplasmic vacuolization	0	6 (100%)	5 (83.3%)	6 (100%)
	I	**–**	1 (16.7%)	**–**
	II	**–**	**–**	**–**
	III	**–**	**–**	**–**
Apoptosis	0	6 (100%)	3 (50%)	2 (33.3%)
	I	**–**	2 (33.3%)	3 (50%)
	II	**–**	**–**	1 (16.7%)
	III	**–**	1 (16.7%)	**–**

0 denotes no lesion, I denotes mild, II denotes moderate, and III denotes severe. Each group has six rats. The percentage in parentheses indicates the proportion of animals in each grade

## Discussion

The lungs are the most vulnerable organ for endogenous oxidants and ROS damage because they interact with the outside environment [35]. In experimental animals, DENA is a common and widely used chemical substance to induce esophageal, hepatic, and pulmonary malignancies [7, 36]. In this study, we evaluated the chemopreventive effect of eugenol on DENA/AAF-induced lung cancer and determined its possible action mechanisms. Our data revealed that eugenol treatment significantly decreased the levels of oxidative stress biomarker (LPO) and inflammatory biomarkers (i.e., TNF-α and IL-1β levels and NF-κB, MCP-1, and NF-κB p65 mRNA expression) and Bcl-2, an antiapoptotic biomarker. In contrast, eugenol treatment significantly increased the GSH content and antioxidant enzyme activities (i.e., GPx and SOD), Nrf2 levels, and apoptotic biomarkers (p53 and Bax mRNA expressions and the Bax/Bcl-2 ratio) compared with those in DENA/AAF-administered control rats.

ROS is involved in tissue cellular signaling, homeostasis, survival, differentiation, and cancer development [37]. In this study, DENA/AAF administration increased the levels of LPO in the lungs and reduced GSH concentration and SOD and GPx activities compared with the normal controls. These findings agree with those of Verma et al. [38] and Li et al. [39], who suggest that DENA is a powerful carcinogen that generates massive amounts of free radicals. The generation of excessive free radicals stimulates lipids to produce LPO, which interacts with DNA to create mutations and ultimately induces carcinogenesis. Furthermore, Sadek et al. [40] proposed that DENA triggered a significant drop in GSH levels and antioxidant enzyme activity, which could be attributed to the excess LPO produced during DENA metabolism. In contrast, eugenol treatment reduced lung LPO levels and increased GSH content and GPx and SOD activities. Our findings agree with those of Choudhury et al. [41], who reported that the reduction in ROS and LPO levels demonstrated eugenol’s chemopreventive activity in the in vivo mouse lung carcinogenesis model. Additionally, Mnafgui et al. [42] reported that eugenol decreased LPO levels while increasing the activities of antioxidative enzyme indicators (i.e., SOD, GPx, and GSH). Eugenol has been shown to reduce the products of lipid peroxidation, indicating that it has antioxidant effects. Furthermore, eugenol enhances the free radical scavenging activity of SOD and GPx enzymes, implying that eugenol has antioxidative properties. Eugenol treatment inhibited the generation of reactive ROS and enhanced the activities of GPx and SOD and GSH content [43]. Eugenol reduces oxidative stress by inhibiting enzymes and oxidative processes, which are associated with its anti-inflammatory drug profile [44]. Moreover, eugenol is known to protect DNA and proteins from oxidative damage, prevent the production of reactive nitrogen species, sift free radicals, increase cyto-antioxidant capacity, and prevent ROS production. In addition, eugenol may repair oxidative damage, remove damaged molecules, and prevent cancer-causing mutations [45]. In contrast, other publication reported that enhancing oxidative stress in cells by producing ROS can selectively trigger tumor cell death pathways, which can remove malignant cells, allowing cancer treatments to be more effective [46].

**Fig. 6:**
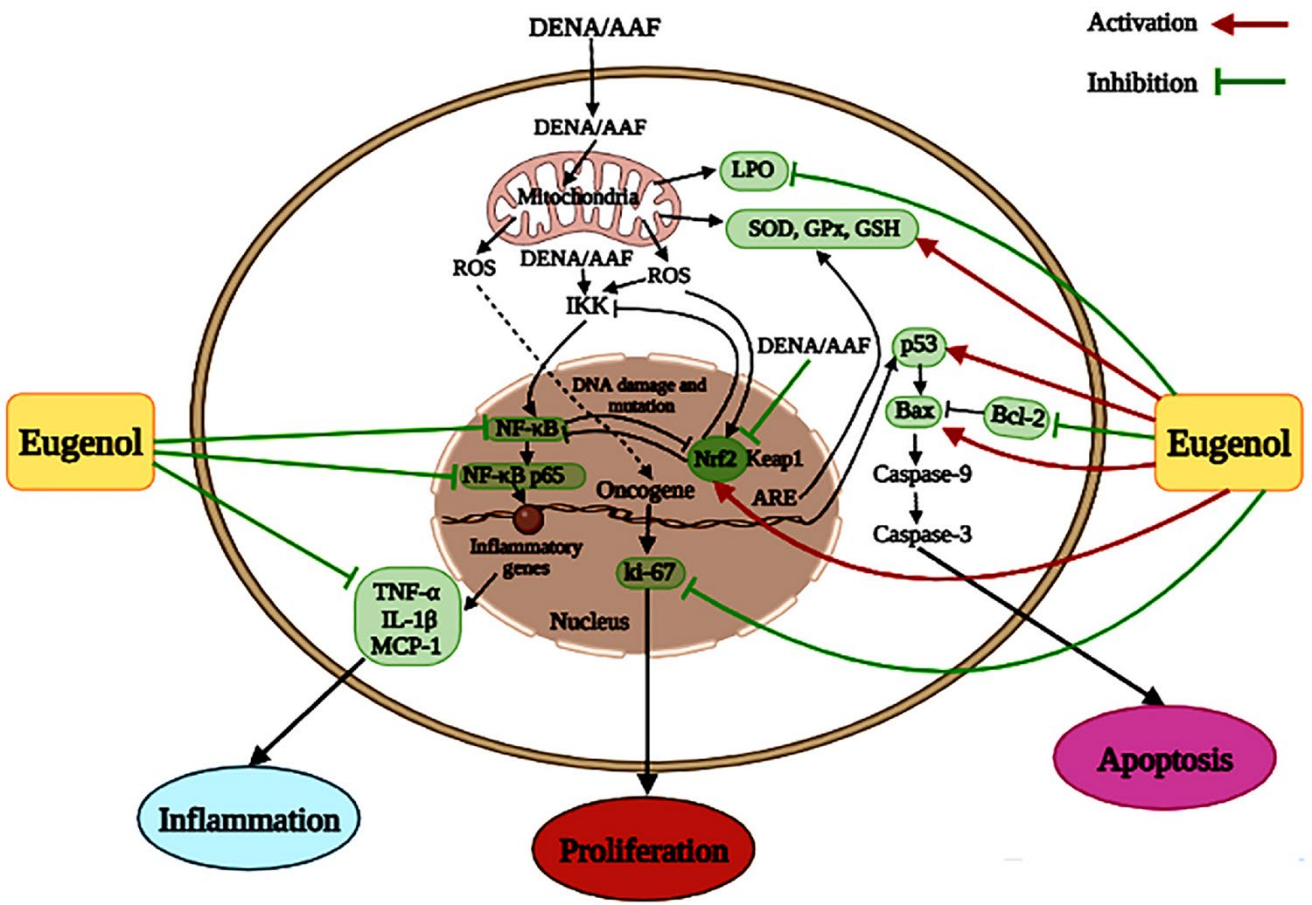
Schematic diagram of eugenol exhibiting antioxidant, antiproliferative, anti-inflammatory, and proapoptotic actions in DENA/AAF-administered rats

Concerning inflammation, this study demonstrated that DENA/AAF administration increased mRNA expression levels of NF-κB and NF-κB p65 relative to normal controls. These results agreed with the findings of Vicentini et al. [47], who suggested that DENA-induced chronic inflammation triggers the release of ROS that activates transcription factors sensitive to oxidative stress, including NF-κB, and proinflammatory transcription factors that cause the release of numerous cytokines (Fig. 6). In the same direction, Tang et al. [48] reported that high levels of NF-κB p65 were found in lung tumors; the nuclear expression of NF-κB p65 is an initial and common feature in lung cancer etiology. In contrast, eugenol treatment reduced the expression levels of NF-κB and NF-κB p65, reflecting its anti-inflammatory effect. In parallel with this result, Abdu et al. reported the anti-inflammatory action of another phenolic, quercetin, in hepatocellular carcinoma was mediated by downregulation of NF-κB [49]. Furthermore, by suppressing NF-κB inhibitory factor degradation and preventing the translocation of NF-κB p65 into the nucleus, eugenol serves as a powerful NF-κB inhibitor, lowering the release of proinflammatory cytokines (Fig. 6) [50]. Eugenol’s anti-inflammatory properties are associated with its molecular mechanism, as evidenced by lower levels of proinflammatory cytokines and inflammation enzyme markers, which are related to redox status modification via reduced LPO and increased antioxidative enzymes. Therefore, our findings strongly suggest that eugenol has chemotherapeutic potential against carcinogenesis [44].

In this investigation, DENA/AAF-administered rats demonstrated a marked rise in the lung levels of TNF-α and IL-1β when compared to the normal controls. These results agreed with those of Bakry et al. [51], who suggested that TNF-α is associated with the development of numerous human disorders, including cancer. In the first week after injection, the higher IL-1β expression was related to a local rise in the proinflammatory cytokine TNF-α and a strong acute inflammatory tissue response with signs of tissue injury. Moreover, eugenol supplementation to DENA/AAF-administered rats exhibited a significant reduction in TNF-α and IL-1β levels. These results are corroborated by those of Abuohashish et al. [52], who observed that the anti-inflammatory activity of eugenol was crucial, as it was followed by a decrease in levels of IL-1β and TNF-α. Eugenol is involved in the transcription of various factors that influence the inflammatory process, including NF-κB, and several cytokines, such as TNF-α and IL-1β [53].

Furthermore, the administration of DENA/AAF elevated MCP-1 levels compared with that in normal controls; these results agreed with those of Pan et al. [54], who proposed that MCP-1 dramatically improved lung cancer detection sensitivity; this biomarker arrangement may be useful for early lung cancer diagnosis, therapy, and prognosis. Moreover, MCP-1 stimulates cancer cell invasion, migration, and proliferation, which contribute to tumor growth [55]. In contrast, the supplementation of eugenol to DENA/AAF-administered rats lowered the increased levels of MCP-1, reflecting the anticancer and anti-inflammatory efficacy of eugenol against lung cancer and further revealing that eugenol may be crucial for reducing lung inflammation and preventing lung cancer. These findings agreed with those of Barboza et al. [44], who suggested that eugenol possesses antioxidant and anti-inflammatory properties; therefore, it may be more beneficial in decreasing inflammation.

In this research, DENA/AAF-administered rats revealed a noticeable decrease in Nrf2 levels in the lungs; this agrees with the findings of Karin and Dhar [56] and de la Vega et al. [57], who proposed that Nrf2 was linked to the pathogenesis, progression, and metastasis of cancer. Nrf2 has long been thought to be a tumor suppressor because its cytoprotective effects are thought to be the primary cellular defense mechanism against both external and internal stresses [58]. Moreover, eugenol treatment in DENA/AAF-administered rats elevated the level of Nrf2. Eugenol enhanced the stabilization and nuclear translocation of Nrf2 (Fig. 6). The stimulation of Nrf2 protects cells from the DNA-damaging effects of ROS and cancer electrophilic chemicals [59].

Apoptotic and antiapoptotic genes, including p53, Bcl-2 and Bax, play a decisive role in apoptosis [60]. Regarding apoptosis, this research demonstrated that the administration of DENA/AAF showed a significant upregulation of the expression of Bcl-2 and downregulated the expression levels of p53 and Bax in the lungs. These findings agreed with the results of Abdel-Moneim et al. [8], who proposed that there was a decline in lung p53 expression level in DENA/AAF-induced rats compared with that in normal control rats. Moreover, the intracellular accumulation of non-functional p53 is associated with an increased risk of various malignancies [61]. P53 is a key element gene that promotes the expression of the Bax gene (Fig. 6). Bax transcription is directly induced and activated by the Bax gene, which increases cell cycle arrest or initiates apoptosis. It has been observed that significant p53 upregulation could indicate that p53 has activated the mitochondrial or intrinsic apoptotic pathway (Fig. 6) [60]. Furthermore, Maharjan et al. [62] proposed that Bax triggers cell death by enhancing the permeability of the mitochondrial outer membrane, inducing an increase in cytochrome C entering the cytoplasm, and lastly activating caspases, resulting in the cleavage of several essential cellular substrates. Furthermore, Bcl-2 reduces apoptosis by decreasing Bax activity (Fig. 6). In contrast, eugenol supplementation significantly upregulated the expression levels of Bax and p53 and downregulated the Bcl-2 expression level. DENA/AAF administration revealed the antiproliferative and anticytotoxic effects of eugenol by inducing cell death via apoptosis; this agrees with that of Manikandan et al. [63], who suggested that eugenol enhances apoptosis and lowers invasion and angiogenesis. Eugenol triggered apoptosis through the mitochondrial pathway via altering Bcl-2 family proteins and angiogenesis [64]. Eugenol induced apoptosis of malignant cells via a process that depends on increased ROS generation and lowered mitochondrial membrane potential, revealing that eugenol may have apoptosis-inducing properties [45]. Apoptosis is essential for regulating organism development, maintaining tissue homeostasis, and preventing cancer [65]. It has distinct morphological features, such as chromatin condensation, cell shrinkage, nuclear fragmentation, membrane blebbing, and apoptotic bodies [46].

The well-known mitochondrial route, which is triggered by the members of the Bcl-2 protein family, which includes Bax and Bcl-2, is a significant apoptotic cell death mechanism. An imbalance in the Bax/Bcl-2 ratio increases cytochrome c levels in the cytoplasm, which then triggers caspase enzymes, which are mostly aided by direct or indirect ROS activity [66]. From our results, we concluded that DENA/AAF administration downregulated the Bax/Bcl-2 ratio by increasing the cell proliferation rate; this result agrees with those of Harandi et al. [60], who suggested that a higher Bax/Bcl-2 ratio results in tumor hypersensitivity to drugs, and an increase in the ratio suggests increased cellular death. In contrast, treatment with eugenol caused significant growth inhibition and apoptosis induction by elevating the Bax/Bcl-2 ratio; this agrees with the findings of Pal et al. [67], who demonstrated eugenol’s ability to modulate the Bax/Bcl-2 ratio. Moreover, the Bax/Bcl-2 ratio is elevated due to increased Bax and decreased Bcl-2 protein expression levels, which in turn causes apoptosis [68]. Moreover, Yoo et al. [69] reported that eugenol promoted ROS accumulation, which changed mitochondrial membrane potential, inducing cytochrome c release and starting a signaling cascade that caused apoptosis.

Regarding proliferative markers, Ki-67 is used as a marker for the proliferation of various tumor cells [70]. This investigation showed that DENA/AAF administration significantly elevated Ki-67 levels; this result agrees with the findings of Castro-Gil et al. [71], who suggested that both DENA and DENA + AAF protocols gradually increased the number of Ki-67-positive cells. Elevated Ki-67 expression enhanced the aggressiveness and invasiveness of lung malignancies [72]. In contrast, DENA/AAF-administered rats treated with eugenol showed a significant reduction in Ki-67 levels due to the antiproliferative and anticancer effects of eugenol; this agrees with the results of Abdullah et al. [73], who found that eugenol has anti-metastatic and antiproliferative activities. Ki-67 may be an effective target in cancer therapy (Fig. 6) [74]. Eugenol functions by inducing ROS generation, which inhibits DNA synthesis and suppresses tumor growth. It has been reported that eugenol reduces tumor size by 40% [75]. Furthermore, Ulanowska and Olas [76] suggested that eugenol can induce the production of intracellular ROS, which can induce cell death by suppressing cell development, disrupting the cell membrane, and destroying DNA.

Concerning histopathological changes, the lungs of DENA/AAF-administered rats showed various malignant lesions, including micropapillary adenocarcinoma, sheets of tumor cells, and apoptotic cells with eosinophilic cytoplasm. These findings agree with those of Abdel-Moneim et al. [8], who mentioned that DENA/AAF primarily induces lung cancer. In this investigation, DENA/AAF-induced lung histological changes yielded a similar result. Moreover, eugenol treatment enhanced different histological lesions. This treatment can minimize oxidative stress caused by DENA/AAF administration and inflammation, increase apoptosis, and reduce cancerous lesions. These findings agree with those of Fawzy et al. [77]. These results showed the precautionary effect of eugenol on DENA/AAF-induced lung cancer.

This study has many limitations, including the estimation of oxidative stress markers (e.g., NO, iNO, CAT, and iNOs). Moreover, the detection of other cytokines (e.g., IL-6, IL-17, IL-23, and IL-37) was one of the study restrictions. This study also lacked the determination of more apoptotic biomarkers (e.g., caspase-3, caspase-8, and caspase-9), which could influence apoptotic processes.

## Conclusion

In conclusion, eugenol has a potent preventive effect on DENA/AAF-induced lung cancer and lung injury, mediated by its anticancer, antioxidant, antiproliferative, anti-inflammatory, antiapoptotic, and anti-metastatic effects (Fig. 6). Based on these findings, this study also provides a promising candidate for lung cancer treatment. Simultaneously, these results further guide clinical studies in investigating the clinical efficacy of eugenol.

## Data Availability

All data generated or analyzed during this study are included in the article.
